# Comparison of French and Worldwide *Bacillus anthracis* Strains Favors a Recent, Post-Columbian Origin of the Predominant North-American Clade

**DOI:** 10.1371/journal.pone.0146216

**Published:** 2016-02-22

**Authors:** Gilles Vergnaud, Guillaume Girault, Simon Thierry, Christine Pourcel, Nora Madani, Yann Blouin

**Affiliations:** 1 Institute for Integrative Biology of the Cell (I2BC), CEA, CNRS, Univ. Paris‐Sud, Université Paris‐Saclay, Gif‐sur‐Yvette, France; 2 Bacterial Zoonoses Unit, Animal Health Laboratory, Anses, University Paris-Est, Maisons-Alfort, France; ContraFect Corporation, UNITED STATES

## Abstract

**Background:**

*Bacillus anthracis*, the highly dangerous zoonotic bacterial pathogen species is currently composed of three genetic groups, called A, B and C. Group A is represented worldwide whereas group B is present essentially in Western Europe and Southern Africa. Only three strains from group C have been reported. This knowledge is derived from the genotyping of more than 2000 strains collected worldwide. Strains from both group A and group B are present in France. Previous investigations showed that the majority of sporadic French strains belong to the so-called A.Br.011/009 group A clade and define a very remarkable polytomy with six branches. Here we explore the significance of this polytomy by comparing the French *B*. *anthracis* lineages to worldwide lineages. We take advantage of whole genome sequence data previously determined for 122 French strains and 45 strains of various origins.

**Results:**

A total of 6690 SNPs was identified among the available dataset and used to draw the phylogeny. The phylogeny of the French B group strains which belongs to B.Br.CNEVA indicates an expansion from the south-east part of France (the Alps) towards the south-west (Massif-Central and Pyrenees). The relatively small group A strains belonging to A.Br.001/002 results from at least two independent introductions. Strikingly, the data clearly demonstrates that the currently predominant *B*. *anthracis* lineage in North America, called WNA for Western North American, is derived from one branch of the A.Br.011/009 polytomy predominant in France.

**Conclusions/Significance:**

The present work extends the range of observed substitution rate heterogeneity within *B*. *anthracis*, in agreement with its ecology and in contrast with some other pathogens. The population structure of the six branches A.Br.011/009 polytomy identified in France, diversity of branch length, and comparison with the WNA lineage, suggests that WNA is of post-Columbian and west European origin, with France as a likely source. Furthermore, it is tempting to speculate that the polytomy’s most recent common ancestor -MRCA- dates back to the Hundred Years' war between France and England started in the mid-fourteenth century. These events were associated in France with deadly epidemics and major economic and social changes.

## Introduction

*Bacillus anthracis*, the causative agent of anthrax, is a spore-forming bacterium present worldwide. The bacterium mainly affects wild and domesticated herbivores, causing a serious, often fatal disease. Spores which are the infectious form of the bacteria can remain in soil for decades before being ingested by animals while browsing or grazing [1, 2]. *B*. *anthracis* possesses, together with *Yersinia pestis*, *Mycobacterium tuberculosis* and a few other major human pathogens, a highly monomorphic and clonal population [3–5]. This may reflect a recent emergence or re-emergence of this pathogen combined with limited opportunities for genetic exchanges.

Since year 2000, major progress has been made in the understanding of the population structure and evolution of the *B*. *anthracis* species owing to the access to whole genome sequence data and the resulting possibility to systematically search for genetic polymorphisms. The first tool used to characterize the species at a large scale was tandem repeats polymorphisms [6, 7]. Multiple Loci Variable Number of Tandem Repeats (VNTR) Analysis (MLVA) has been applied to more than two thousand strains worldwide (reviewed in [8]). Most of the associated published data can be queried via databases accessible on the internet [8, 9].

The resulting view of the population structure of *B*. *anthracis* was used to select representative strains for whole genome sequencing and search for single nucleotide polymorphisms (SNPs). A set of 14 single nucleotide polymorphisms (called canonical SNPs or canSNPs) able to subdivide all isolates into the three major lineages (A, B and C) and 13 sub-lineages or sub-groups [10–14], consistent with the genetic diversity previously uncovered by applying MLVA, was proposed. Additional SNPs that define the A.Br.Ames and A.Br.WNA lineages or that lie on various branches of the *B*. *anthracis* SNP tree have been investigated [11, 14–17] to address more specific points.

Whole genome SNP surveys allowed proposing age estimates for key evolutionary steps in the evolutionary history of *B*. *anthracis*. The most recent common ancestor (MRCA) of the A, B and C lineages is estimated to be a few tens of thousands years old [11].

In spite of these major achievements in the past 15 years, information on the geographic origin of *B*. *anthracis*, and on time-scales regarding the more recent evolution events is still lacking. One exception is the proposed dating of the spread of the lineage which is largely dominant in North America (the Western North America or WNA lineage). The genetic diversity observed along this lineage suggested an origin via the Bering land bridge well before the arrival of European colonizers [15, 18]. The analyses we present in this report challenge this interpretation.

Whole genome SNP analysis is an unbiased genotyping tool giving very high resolution with strong phylogenetic content. Owing to rapid progress in this field, the approach can now be applied at a reasonable cost especially since the number of *B*. *anthracis* isolates is very limited in comparison with pathogens of much higher prevalence. We previously reported the MLVA typing, canSNP characterization and whole genome SNP analysis of 122 strains of *B*. *anthracis* isolated in France in the past decades ([8, 17, 19]). French strains (mostly veterinary isolates with robust geographic assignment) resolved into about thirty MLVA genotypes belonging to one of the three canSNP lineages, B.Br.CNEVA, A.Br.011/009 and A.Br.001/002. In particular we described that the A.Br.011/009 lineage is a remarkable polytomy with six branches radiating from its MRCA. In the present report, taking advantage of additional published draft or complete whole genome sequences we describe in detail and discuss the phylogenetic position of the French strains within the larger *B*. *anthracis* phylogeny. In particular, we demonstrate that the WNA lineage emerged from the A.Br.011/009 polytomy. Consequently, and taking into account historical events, we argue that the WNA lineage is a recent lineage resulting from a post-columbian introduction from Europe, followed by a strongly accelerated evolution rate.

## Materials and Methods

### Whole genome sequence (WGS) and data analysis

The strains used are listed in S1 Table. Data analysis from the french strains was previously described by Girault et al, 2014 [17, 20]. Seven reference strains are from the Pasteur Institute’s collection, CIP. Among these, CIP 74.12 is a representative of the Pasteur II strain used in the Pouilly-le-Fort vaccine demonstration in 1881 and CIP A204 was also isolated in the Pasteur laboratory presumably before the creation of the Pasteur Institute in 1888. The other 115 strains were collected during animal or human anthrax outbreaks (mostly from bovine origin) that have occurred in France over the past 31 years (1982–2013). Additional genome sequence data was downloaded from Genbank: Sterne (NC_005945.1), CDC684 (NC_012581.1, [21]), A0248 (NC_012659.1), H9401 (NC_017729.1, [22]), A16R (CP001974.1), SVA11 (CP006742.1), HYU01 (CP008846.1), Vollum (CP007666.1), CNEVA-9066 (French strain, B-group, bioproject PRJNA54133), A1055 (bioproject PRJNA54131), Kruger_B (bioproject PRJNA54105), Western North America USA6153 (bioproject PRJNA54107), Australia_94 (bioproject PRJNA54137), Tsiankovskii-I (bioproject PRJNA54481), Carbosap (bioproject PRJNA224116, [23]), Heroin Ba4599 (bioproject PRJNA190302), UR-1 (bioproject PRJNA180871 [24]), BF-1 (bioproject PRJNA180907, [25]), Sen2Col2, Sen3, Gmb1 (bioprojects PRJEB1516, PRJEB1517, PRJEB1518, [26]), V770-NP1-R (bioproject PRJNA224116, [27]), A0442 (bioproject PRJNA54997), A0488 (bioproject PRJNA54995), 3154 and 3166 (bioprojects PRJNA198411 and PRJNA198412, [28]), A0174 (bioproject PRJNA55003), A0193 (bioproject PRJNA55005), A0389 (bioproject PRJNA54999), 95014 (French strain, A-group, bioproject PRJNA224116), and three Georgian strains (bioprojects PRJNA224563, PRJNA224558, and PRJNA224562, [29]).

Ames Ancestor (GenBank accession no. NC_007530.2) was used as the reference genome for assembly. SNPs identification was done as previously described [30] using BioNumerics version 7.5 (Applied Maths, Belgium). Sets of pseudo-reads of 100 bp length were created *in silico* when using contigs or whole genome data. A set of SNPs was deduced for each genome sequence data using the BioNumerics Chromosome Comparisons module. SNP positions at which one or more strains displayed an ambiguous residue call or missing data were discarded. Ribosomal operons and repeated sequences including tandem repeats were masked. The list of SNPs positions is provided in S2 Table.

### Whole Genome Phylogenetic Analysis

A minimum spanning tree was drawn in BioNumerics by using the filtered whole genome sequencing SNP data as input. The tree was rooted by using *B*. *cereus* strain AH820 (accession number NC_011773) [31]. The *B*. *cereus* genome was interrogated for each SNP position identified within *B*. *anthracis*. The canSNPs positions in the global tree were identified from the list of SNPs (S2 Table).

## Results and Discussion

### Whole Genome Sequencing and SNP identification

**Fig 1:**
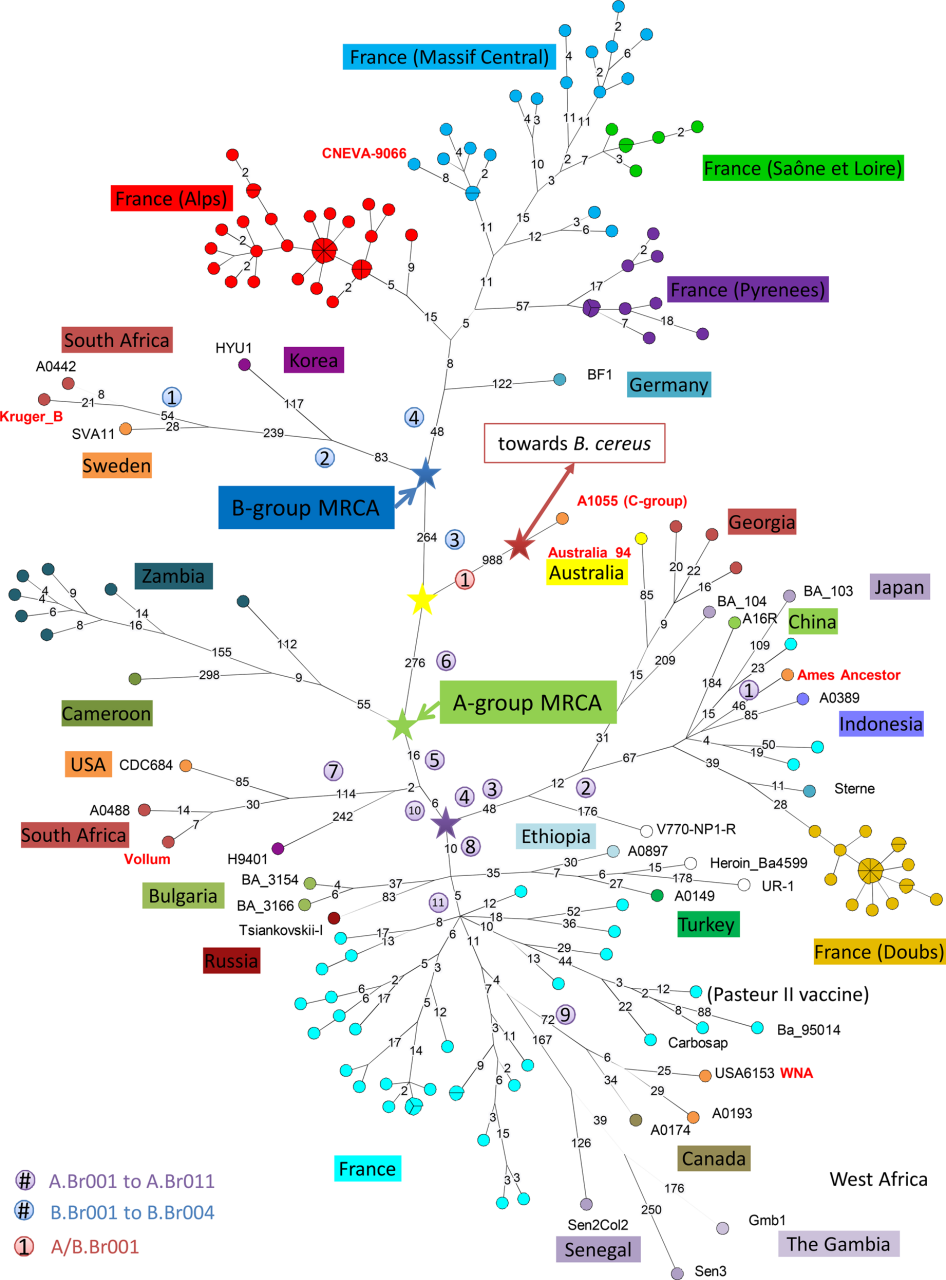
Minimum spanning tree of 167 *B*. *anthracis* strains based on whole-genome SNP analysis. Data are based on 6690 chromosomal SNPs. Geographic origin is reflected by a color code shown on the figure. Each circle represents a unique SNP genotype, the diameter varies according to the number of isolates having the same genotype. The length of each branch is proportional (logarithmic scale) to the indicated number of SNPs. Branches devoid of length value indication have a length of one SNP. The name of the published genomes included in this study is indicated next to their node in the tree. Five colored stars mark major splits in the *B*. *anthracis* phylogeny. CanSNPs are indicated in colored disks next to the branch where they occur in the tree.

A total of 6690 chromosomal SNPs was identified by mapping sequence data from 166 strains (122 French strains and 44 additional strains of worldwide origin) against the Ames ancestor reference genome. Fig 1 illustrates the minimum spanning tree (MST) deduced from the SNP data. The color code reflects geographic origins and the strain Id of the 45 additional strains (including the Ames strain) is indicated. The position of the root, as determined by projecting the *Bacillus cereus* strain is indicated by the red star. In agreement with previous analyses [10], the root is located along the branch leading to the lineage C representative. This root represents the position of the MRCA of the currently known *B*. *anthracis* lineages A, B and C. The yellow star indicates the position of the MRCA of the A and B lineages. The distance from the yellow star to tips is comparable along lineages A and B. Along lineage A, the maximum distance corresponds to the west African strain Sen3 [26] located at a distance of 794 SNPs. The shortest branch corresponds to a French isolate located at a distance of 325 SNPs from the yellow star. Along lineage B, strain Kruger_B is located at a distance of 661 SNPs whereas some isolates from the French Alps are located at a distance of 340 SNPs. The most recent common ancestor (MRCA) of the A (green star) and the B (blue star) lineages occur at an almost identical distance from the split between the two lineages indicated by the yellow star (276 versus 264). From these two points, the shortest branches extend for a further 49 and 76 SNPs whereas the longest extend for 518 and 397 SNPs respectively. The two longest branches occur in Africa (Senegal and South Africa), whereas the two shortest are represented by French isolates. This ratio of up to ten between average substitution rate along different lineages is much higher than the previously observed ratio of three [10].

The seven genomes which were initially used to define canSNPs are indicated in red. The position of each of the 16 canSNPs (including A.Br.005, A.Br.010 and A.Br.011 which are not part of the standard and most used canSNPs assay) is indicated by numbers in colored circles, with a different color code for each of the three lineages, A, B and C [11, 32]. In agreement with previous reports [8, 17, 19, 33], the French isolates clustered into three canSNP types: A.Br.001/002 (branches located between canSNPs A.Br.001 and A.Br.002), A.Br.011/009 (branches located between canSNPs A.Br.009 and A.Br.011) and B.Br.CNEVA (branches beyond canSNP B.Br.004). The most basal node within group A is defined by the split of the A.Br.005/006 canSNP type represented by one strain from Cameroon [17] and seven from Zambia [34, 35]. The Cameroon strain was previously shown to belong to MLVA cluster E described by Lista et al. [36] corresponding to the "?" cluster described by Le Flèche et al. [7] and to the basal Aß lineage described by Maho et al. [37]. This lineage is also widely represented in Chad as can be seen by querying MLVAbank. Fig 1 illustrates that if A.Br.005 is not assayed, as is the case in most canSNPs investigations, a strain such as Korea H9401 formally belonging to A.Br.005/007 corresponding to MLVA cluster D in [36] might be wrongly assigned to A.Br.005/006. The more relaxed A.Br.006/007 canSNP designation should be used in the absence of A.Br.005 typing [38]. Consequently, published assignments to A.Br.005/006 lineage should be considered with caution unless A.Br.005 has been typed. For instance, the A.Br.005/006 canSNP type strains in van Ert et al. [11] are instead most likely A.Br.005/007 according to their MLVA clustering in close proximity with the A.Br.Vollum lineage, characterized by a derived nucleotide for the A.Br.007 SNP position.

The purple star in Fig 1 indicates the position of the MRCA of lineages leading to two extensively studied canSNP types, A.Br.WNA and A.Br.Ames. A.Br.WNA is located beyond three canSNPs occurring in the order A.Br.008, A.Br.011, and A.Br.009. A.Br.Ames is located after A.Br.004, A.Br.003, A.Br.002 and A.Br.001. Distances from the purple star to the tips towards these two directions differ greatly. Whereas distances of 131 to 300 SNPs are observed towards A.Br.Ames, a minimum of 27 and a maximum of 491 are observed towards A.Br.WNA.

### A.Br.008/011, A.Br.011/009 and A.Br.WNA phylogenetic analysis

The A.Br.008/011 lineage is represented by seven strains, including two strains from Bulgaria [28], one representative of the historical Russian vaccine strain Tsiankovskii-I, two strains of unknown geographic origin recovered from drug users [13, 24], one strain from Turkey, and one from Ethiopia. The two strains isolated from drug users, UR-1 and Ba4599 [13, 24], are very closely related but clearly distinct. UR-1 is characterized by a relatively long branch (178 SNPs) as compared to the Ba4599 terminal branch (15 SNPs). Although not formally impossible in view of other substitution rate distortions, the long branch would need to be confirmed by using the raw Ion Torrent data rather than the resulting available WGS contig assemblies as done in the present investigation. The Ion Torrent technology has been shown to have a higher error rate [39] and random sequencing errors will increase the length of terminal branches. If the UR-1 strain is not taken into account, the length from the A.Br.008/011 group MRCA to the tips is similar, 42–44 SNPs towards the two Bulgarian strains, 84 towards the Tsiankovskii-I Russian vaccine strain, 63 towards the second drug user strain of unknown geographic origin, 65 and 69 towards the strains from Ethiopia and Turkey respectively. The slightly longer vaccine strain branch might reflect extensive *in vitro* cultivation. These seven A.Br.008/011 strains define two branches starting from the group MRCA. A third branch contains canSNP A.Br011 and leads to the A.Br.011/009 group comprising the majority of sporadic French strains.

The A.Br.011/009 strains define a polytomy with six branches radiating from a central node located at 15 SNPs from the purple star (Fig 1) [17]. The distances from this central node are very variable. They range from a minimum of 12, up to 471 SNPs, corresponding to a remarkable ratio of almost 40. The longest branches are produced by strains from Senegal and Gambia [26]. Even if terminal branches which may concentrate sequencing errors are not taken into account, the length ratio is still 12 to 221, i.e. 18. Very interestingly, the WNA lineage located exclusively in North America [11, 15] and represented here by three strains, one from Canada and two from the United States represent the second longest branch, 120–124 SNPs, a ratio of 10 compared to the shortest branch. The WNA and the Senegal-Gambia lineages split very early after departing from the French lineages.

**Fig 2:**
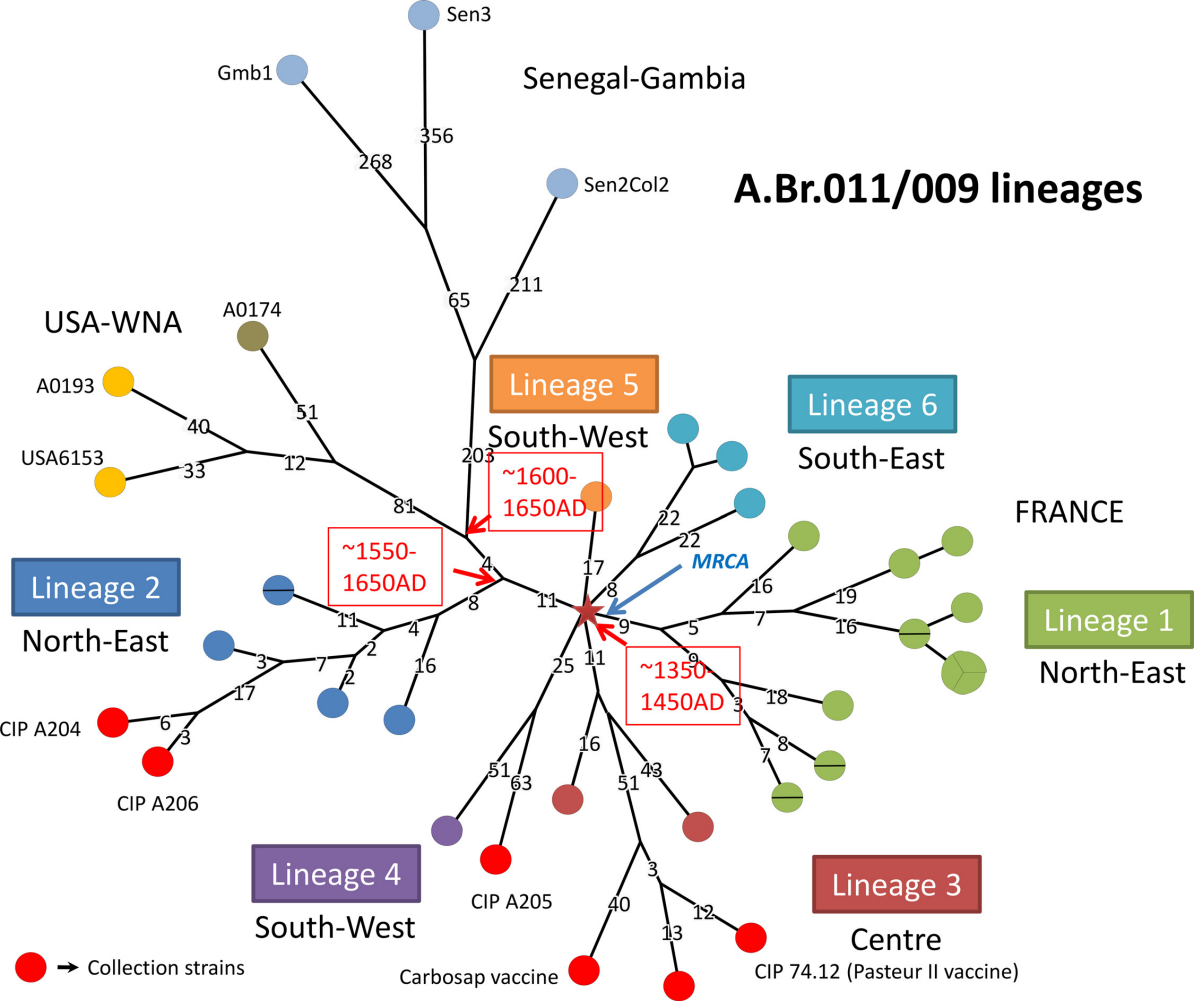
Minimum spanning tree of 38 *B*. *anthracis* strains belonging to the A.Br.011/009 polytomy. Tree drawing is based upon 1934 chromosomal SNPs. The six branches constituting the polytomy are numbered as previously reported [17]. The diameter of each circle varies according to the number of isolates having the same genotype. The length of each branch is proportional (logarithmic scale) to the indicated number of SNPs. Branches with no indication of length value have a length of one SNP. A tentative dating of the initial outbreak represented by the red star is indicated, based on the assumption that (1) the split towards WNA-Senegal-Gambia occurred within 1550–1650 (2) the average expansion rate of lineage 2 which remained in the same ecosystem (within France) did not vary from the founder outbreak event until the end of the nineteenth century. Under these hypotheses, the outbreak most likely occurred during the hundred years war between France and England, 1350–1450.

In order to maximize the resolution of the SNP analysis, the SNP screen was applied to the subset of A.Br.011/009 and A.Br.WNA strains. Less SNPs are excluded when less strains are taken into account, mostly due to deletions, partial genome assemblies in public WGS data and/or sequence quality issues in some data sets. Within this subset of strains, 1934 SNPs are identified as compared to 1451 in the corresponding portion of Fig 1, and used to draw Fig 2. The lineages are numbered from 1 to 6 as in [17]. The length of the different French lineages is highly variable, from 17 for lineage 5 up to 76 for lineage 4 not taking into account French strains obtained from third parties–collections potentially more extensively cultured. The red star indicates the position of the root. The WNA and Senegal-Gambia lineages are branching out from lineage 2 at a distance of 11 SNPs from the central node of the polytomy. Only four SNPs later, WNA and Senegal-Gambia split. Lineage WNA extends for a further 133 SNPs, and Senegal-Gambia for 624 SNPs (or 268 if not taking into account the terminal segments) whereas the rest of lineage 2 extends for only 16 up to 24 SNPs when not taking into account two collection strains. This demonstrates that within the same time span and among very closely related lineages, substitution rates can differ by a ratio of more than 15, i.e. much higher than previously reported. The WNA-Senegal-Gambia split occurs at 30% to 40% along the lineage 2 expansion.

Along this line, the presence in this polytomy of a very short branch (17 SNPs for the lineage 5) found in France has a great impact on previous propositions for the evolution of *B*. *anthracis*. The previously proposed pre-columbian Bering Strait hypothesis for the importation of the WNA branch in North America was prompted by the very long branch length of the WNA lineage [15]. The present analysis clearly demonstrates that there is no need to invoke an ancient origin for the WNA lineage. In the same time-span, lineage 5 reached a length of only 17 SNPs, compared to the 151 SNPs length for the WNA lineage. In other words, 151 SNPs are not indicative of a longer time-span than 17 SNPs. A more likely interpretation for the fast expansion of the WNA and Senegal-Gambia lineages is that they occurred in an ecosystem which was new to the infection by *B*. *anthracis*, was highly sensitive, and in which *B*. *anthracis* benefited from a much increased number of generations per year.

### A.Br.001/002 and A.Br.Ames phylogenetic analysis

**Fig 3:**
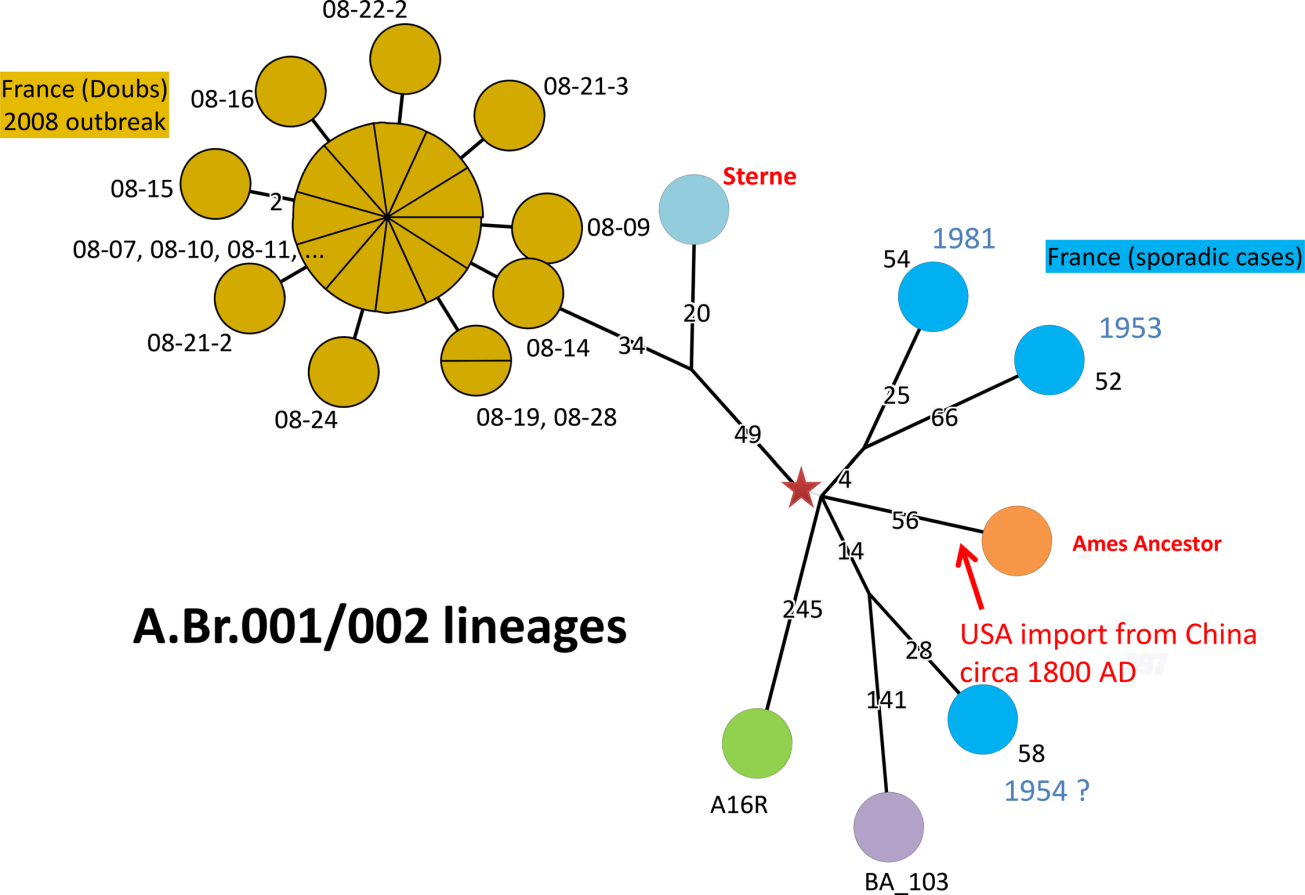
Minimum spanning tree of 28 *B*. *anthracis* strains belonging to the A.Br.001/002 polytomy. The tree is based upon 1669 chromosomal SNPs. The diameter of each circle varies according to the number of isolates sharing the same genotype. The length of each branch is proportional (logarithmic scale) to the indicated number of SNPs. Branches with no indication of length value have a length of one SNP. The red star indicates the position of the root, as defined by using the Australia_94 strain genome as an outgroup. The red arrow indicates the approximate position of the timing of the introduction of the Ames lineage to the USA according to [14].

Sequence analysis identified 231 chromosomal SNPs that differentiate the 24 A.Br.001/002 French strains. The length from the purple star to the tips is similar and varies from 131 for one Georgian strain to 198 (Fig 1). All 21 strains from the Doubs *department* formed a single clonal cluster differing by a maximum of two SNPs and they belong to the same branch as the Sterne vaccine strain. They will together be subsequently called the Sterne lineage. The three additional A.Br.001/002 strains are older samples (collected in 1953, 1954 and 1981) isolated in other *departments* of France. They are clearly unrelated to the recent Doubs outbreak [40] strains and contribute to a polytomy with four branches, one of which is leading to the A.Br.Ames sublineage (Fig 1). Fig 3 provides a focus on the A.Br.001/002 polytomy. The length of the four branches varies from a minimum of 29 SNPs to a maximum of 245 (ratio of 8). The branching point of the Sterne lineage is only one SNP away from the root of the A.Br.001/002 polytomy. This is quite similar to the A.Br.011/009 polytomy and typical of an outbreak, as illustrated here by the Doubs outbreak. All four A.Br.001/002 lineages together with the A.Br.Ames lineage appear to result from a single outbreak event. Simonson et al. [14] investigated 191 *B*. *anthracis* isolates from China, 74 of which were canSNP typed as A.Br.001/002 and 8 were A.Br.Ames. The authors estimate from historical records that the Ames lineage present in the Texas/Louisiana Gulf region split from its Chinese progenitor lineage in the early to middle 1800s. Since the USA-import, the USA specific Ames lineage expanded between 4 and 12 SNPs. The most part of the Ames lineage expansion occurred while in China, before the USA-import.

### The tentative dating of the A.Br.011/009 polytomy, and of its most recent common ancestor

Under the hypothesis of a French emergence of the A.Br.011/009 polytomy, as implied by the very short French branch (12 SNPs in Fig 1), *B*. *anthracis* would have been introduced to North America from Europe, perhaps France. The WNA lineage is believed to have been present in North America at least since 1770: anthrax was present in Haiti at that time according to historical records [41, 42] and the WNA lineage is present in Haiti [11]. We hypothesize that the split leading to WNA corresponds to an introduction of anthrax in North America via French settlers to Quebec or other French colonies in the beginning of the seventeenth century. Interestingly, the WNA lineage and the Africa lineage are splitting early (4 SNPs) after the emerging from the lineage 2 identified in France. This short distance seems consistent with a split at the same period of time and with the hypothesis that French navigators could have introduced *B*. *anthracis* strains in others countries. French settlements were established in Eastern North America (“Nouvelle France”) since the beginning of the seventeenth century (Quebec, 1608) and in Western Africa because of the slave trade almost at the same period (1600–1650). The trade of textiles could have been a source of introduction of the disease in North America and Africa. Later on, the better understanding of the disease, and the strong prophylactic and control measures applied in France since the end of the nineteenth century have probably slowed down the turn-around of *B*. *anthracis* during the twentieth century. We can consequently reasonably hypothesize that the French lineage kept expanding at an unaffected rate after the WNA split for approximately 300 years i.e. from year 1600 until 1900 corresponding to two-thirds of the French lineage length. 1300–1500 is consequently a likely estimate for the dating of the outbreak which gave birth to the A.Br.011/009 polytomy. In France, years 1350–1450 corresponds to the "One hundred years war" with England (1337–1453). It was characterized by major epidemics (including the Black Death plague due to *Yersinia pestis*), civil war events, and major economic changes. At that time, South-West France was occupied by England. Uncontrolled armed groups were travelling across large regions of France. The associated events would have favored the geographic spreading and subsequent fixation across most of France. This period is thus a good candidate for the dating of the root of the A.Br.011/009 polytomy observed in France.

### Cluster B and B.Br.CNEVA phylogenetic analysis

More than half of the *B*. *anthracis* isolates from France clustered within the B2 B.Br.CNEVA lineage. The MLVA sub-division of cluster B in sublineages B1 and B2 [6] including respectively B.Br.Kruger and B.Br.CNEVA [11] is clearly visible. The length from the MRCA of cluster B strains to the tips is highly variable. It varies from 77 up to 160 within the B2 cluster representatives, as compared to the 426 SNPs to South African “Kruger B”. This corresponds to a ratio of more than 5, higher than the previous estimate of 3 [10] and this increase is due to the inclusion of the Alps lineage. Starting from the MRCA of the French Cluster B strains, the Pyrenees lineage which includes the A0465 strain shows the longest branch (range 71–92 SNPs) whereas the Alps lineage is the shortest (range 21–26 SNPs, ratio is approximately 1/3). The inclusion of the lineage leading to BF1 defines an older MRCA for lineage B2 (Fig 1). BF1 originates from Bavaria in Germany, which contains part of the Alps [25]. This tree topology suggests that the spreading of *B*. *anthracis* to the Massif-Central and the Pyrenees represents secondary events and spill-outs from the Alps. The faster rate of evolution observed in the Pyrenees is in agreement with the previous suggestion that *B*. *anthracis* evolutionary rate accelerates significantly as new territories with naïve populations are encountered [10, 11]. Apart from the polytomies with very short branches -1 or 2 SNPs- associated with the typical outbreak observed in the Alps strains, no significant polytomy is observed in the whole B-group.

### Mutations in vaccine strains

Collection strains extend lineage 2, lineage 3 and lineage 4 in the A.Br.011/009 polytomy by 26, 52 and 63 SNPs respectively. Lineage 3 is of special interest as it contains two well-known vaccine strains, the Pasteur II strain and one of its derivatives, Carbosap widely used in Italy (these vaccine strains are pXO1+, pXO2+). In agreement with these historical records, the Carbosap branch is the longest presumably as a result of more extensive cultivation. Three chromosomal deletions of 29, 24 and 3.5 kb relative to virulent strains have been described in Carbosap [23]. Interestingly, the 3.5 kb deletion, at position 1235796–1239220, including locus tags GBAA_1286 to GBAA_1290 in Ames ancestor NC_007530.2 and 24 kb deletion at position 1640915–1665378, GBAA_1751-GBAA_1781 including the virulence associated gene GBAA_1760 [31] are already present in the Pasteur II strain whereas the third region (position 267162–294257) is intact in Pasteur II. Pasteur II, Carbosap and Tsiankovskii I are vaccine strains possessing both virulence plasmids, so deletions in these strains may be associated with loss of virulence factors.

## Conclusions

In this report, we position the genetic diversity found within the *B*. *anthracis* population in France in the broader context of the worldwide diversity of this species. France is one among a few countries where both the A and B branches are represented [11]. The A.Br.011/009 lineage is the most wide-spread in France and constitutes a remarkable polytomy with six branches. The present report shows that the WNA lineage predominent in North America belongs to this polytomy. The large length of the WNA branch prompted previous investigators to propose a pre-Columbian origin for this lineage. The analysis of the A.Br.011/009 polytomy demonstrates instead that this length is due to a remarkable acceleration of the evolution of the WNA branch after the split from the progenitor lineage. In the same time-span, the average pace of expansion of the progenitor lineage was slower by a factor of ten. The speed of expansion of the Senegal-Gambia lineage was even faster. Van Ert et al [11] previously proposed that the evolution rate of *B*. *anthracis* would be faster after introduction in a previously *B*. *anthracis* free environment. This would fit with the present observations, and, conversely, suggest that lineage expansion rates may provide indications on the origin of a lineage. In this view, short lineages (less successful, lower number of generations per year, rare) would tend to be closer to their birth place. Because of the recent introduction of A.Br.011 SNP typing, the distribution of the A.Br.011/009 lineage in the world is poorly known. In order to test the hypotheses proposed here, it will be necessary to more precisely investigate the presence and genome sequence of A.Br.011/009 in neighboring European countries and elsewhere.

## Supporting Information

S1 Table List of strains used in this study. The geographic origin and year of isolation are indicated when known.
(XLSX)

S2 Table General list of SNPs and genotypes of strains used to draw Fig 1. The position given refers to Ames ancestor genome accession number NC_007530.2. canSNPs are indicated.
(XLSX)

## References

[1] Hugh-Jones M, Blackburn J. The ecology of *Bacillus anthracis*. Mol Aspects Med. 2009;30(6):356–67. Epub 2009/09/02. 10.1016/j.mam.2009.08.003 S0098-2997(09)00061-2 [pii]. . 19720074

[2] Revich BA, Podolnaya MA. Thawing of permafrost may disturb historic cattle burial grounds in East Siberia. Glob Health Action. 2011;4 Epub 2011/11/25. 10.3402/gha.v4i0.8482 GHA-4-8482 [pii]. 22114567 PMC3222928

[3] Achtman M. Evolution, population structure, and phylogeography of genetically monomorphic bacterial pathogens. Annu Rev Microbiol. 2008;62:53–70. Epub 2008/09/13. 10.1146/annurev.micro.62.081307.162832 . 18785837

[4] Blouin Y, Cazajous G, Dehan C, Soler C, Vong R, Hassan MO, et al Progenitor "*Mycobacterium canettii*" clone responsible for lymph node tuberculosis epidemic, Djibouti. Emerg Infect Dis. 2014;20(1):21–8. Epub 2014/02/13. 10.3201/eid2001.130652 24520560 PMC3884719

[5] Cui Y, Yu C, Yan Y, Li D, Li Y, Jombart T, et al Historical variations in mutation rate in an epidemic pathogen, *Yersinia pestis*. Proc Natl Acad Sci U S A. 2013;110(2):577–82. Epub 2012/12/29. 10.1073/pnas.1205750110 1205750110 [pii]. 23271803 PMC3545753

[6] Keim P, Price LB, Klevytska AM, Smith KL, Schupp JM, Okinaka R, et al Multiple-locus variable-number tandem repeat analysis reveals genetic relationships within *Bacillus anthracis*. J Bacteriol. 2000;182(10):2928–36. 10781564 10.1128/jb.182.10.2928-2936.2000 PMC102004

[7] Le Flèche P, Hauck Y, Onteniente L, Prieur A, Denoeud F, Ramisse V, et al A tandem repeats database for bacterial genomes: application to the genotyping of *Yersinia pestis* and *Bacillus anthracis*. BMC Microbiol. 2001;1(1):2. 11299044 10.1186/1471-2180-1-2 PMC31411

[8] Thierry S, Tourterel C, Le Flèche P, Derzelle S, Dekhil N, Mendy C, et al Genotyping of French *Bacillus anthracis* Strains Based on 31-Loci Multi Locus VNTR Analysis: Epidemiology, Marker Evaluation, and Update of the Internet Genotype Database. PLoS One. 2014;9(6):e95131 Epub 2014/06/06. 10.1371/journal.pone.0095131 PONE-D-13-27818 [pii]. 24901417 PMC4046976

[9] Grissa I, Bouchon P, Pourcel C, Vergnaud G. On-line resources for bacterial micro-evolution studies using MLVA or CRISPR typing. Biochimie. 2008;90(4):660–8. . 17822824 10.1016/j.biochi.2007.07.014

[10] Pearson T, Busch JD, Ravel J, Read TD, Rhoton SD, U'Ren JM, et al Phylogenetic discovery bias in *Bacillus anthracis* using single-nucleotide polymorphisms from whole-genome sequencing. Proc Natl Acad Sci U S A. 2004;101(37):13536–41. Epub 2004/09/07. 10.1073/pnas.0403844101 0403844101 [pii]. 15347815 PMC518758

[11] Van Ert MN, Easterday WR, Huynh LY, Okinaka RT, Hugh-Jones ME, Ravel J, et al Global genetic population structure of *Bacillus anthracis*. PLoS One. 2007;2(5):e461 Epub 2007/05/24. 10.1371/journal.pone.0000461 17520020 PMC1866244

[12] Marston CK, Allen CA, Beaudry J, Price EP, Wolken SR, Pearson T, et al Molecular epidemiology of anthrax cases associated with recreational use of animal hides and yarn in the United States. PLoS One. 2011;6(12):e28274 Epub 2011/12/17. 10.1371/journal.pone.0028274 PONE-D-11-08570 [pii]. 22174783 PMC3235112

[13] Price EP, Seymour ML, Sarovich DS, Latham J, Wolken SR, Mason J, et al Molecular epidemiologic investigation of an anthrax outbreak among heroin users, Europe. Emerg Infect Dis. 2012;18(8):1307–13. Epub 2012/07/31. 10.3201/eid1808.111343 22840345 PMC3414016

[14] Simonson TS, Okinaka RT, Wang B, Easterday WR, Huynh L, U'Ren JM, et al *Bacillus anthracis* in China and its relationship to worldwide lineages. BMC Microbiol. 2009;9:71 Epub 2009/04/17. 10.1186/1471-2180-9-71 1471-2180-9-71 [pii]. 19368722 PMC2674057

[15] Kenefic LJ, Pearson T, Okinaka RT, Schupp JM, Wagner DM, Hoffmaster AR, et al Pre-Columbian origins for North American anthrax. PLoS One. 2009;4(3):e4813 Epub 2009/03/14. 10.1371/journal.pone.0004813 19283072 PMC2653229

[16] Birdsell DN, Pearson T, Price EP, Hornstra HM, Nera RD, Stone N, et al Melt analysis of mismatch amplification mutation assays (Melt-MAMA): a functional study of a cost-effective SNP genotyping assay in bacterial models. PLoS One. 2012;7(3):e32866 Epub 2012/03/23. 10.1371/journal.pone.0032866 PONE-D-11-21413 [pii]. 22438886 PMC3306377

[17] Girault G, Blouin Y, Vergnaud G, Derzelle S. High-throughput sequencing of *Bacillus anthracis* in France: investigating genome diversity and population structure using whole-genome SNP discovery. BMC Genomics. 2014;15(1):288 Epub 2014/04/17. 10.1186/1471-2164-15-288 1471-2164-15-288 [pii]. 24734872 PMC4023602

[18] Kenefic LJ, Okinaka RT, Keim P. Population genetics of *Bacillus*: phylogeography of anthrax in North America In: Robinson DA, Falush D, Feil EJ, editors. Bacterial population genetics in infectious disease. Hoboken, New Jersey: Wiley-Blackwell; 2010 p. 169–79.

[19] Derzelle S, Laroche S, Le Fleche P, Hauck Y, Thierry S, Vergnaud G, et al Characterization of genetic diversity of *Bacillus anthracis* in France by using high-resolution melting assays and multilocus variable-number tandem-repeat analysis. J Clin Microbiol. 2011;49(12):4286–92. Epub 2011/10/15. 10.1128/JCM.05439-11 21998431 PMC3232934

[20] Girault G, Parisot N, Peyretaillade E, Peyret P, Derzelle S. Draft Genomes of Three Strains Representative of the *Bacillus anthracis* Diversity Found in France. Genome Announc. 2014;2(4). Epub 2014/08/02. 10.1128/genomeA.00736-14 e00736-14 [pii]2/4/e00736-14 [pii]. 25081258 PMC4118061

[21] Okinaka RT, Price EP, Wolken SR, Gruendike JM, Chung WK, Pearson T, et al An attenuated strain of *Bacillus anthracis* (CDC 684) has a large chromosomal inversion and altered growth kinetics. BMC Genomics. 2011;12:477 Epub 2011/10/04. 10.1186/1471-2164-12-477 1471-2164-12-477 [pii]. 21962024 PMC3210476

[22] Chun JH, Hong KJ, Cha SH, Cho MH, Lee KJ, Jeong DH, et al Complete genome sequence of *Bacillus anthracis* H9401, an isolate from a Korean patient with anthrax. J Bacteriol. 2012;194(15):4116–7. Epub 2012/07/21. 10.1128/JB.00159-121 94/15/4116 [pii]. 22815438 PMC3416559

[23] Harrington R, Ondov BD, Radune D, Friss MB, Klubnik J, Diviak L, et al Genome Sequence of the Attenuated Carbosap Vaccine Strain of *Bacillus anthracis*. Genome Announc. 2013;1(1). Epub 2013/02/14. 10.1128/genomeA.00067-12 e00067-12 [pii]genomeA00067-12 [pii]. 23405332 PMC3569327

[24] Rückert C, Licht K, Kalinowski J, Espirito Santo C, Antwerpen M, Hanczaruk M, et al Draft genome sequence of *Bacillus anthracis* UR-1, isolated from a German heroin user. J Bacteriol. 2012;194(21):5997–8. Epub 2012/10/10. 10.1128/JB.01410-12 194/21/5997 [pii]. 23045504 PMC3486082

[25] Antwerpen M, Proenca DN, Ruckert C, Licht K, Kalinowski J, Hanczaruk M, et al Draft genome sequence of *Bacillus anthracis* BF-1, isolated from Bavarian cattle. J Bacteriol. 2012;194(22):6360–1. Epub 2012/10/30. 10.1128/JB.01676-12 194/22/6360 [pii]. 23105087 PMC3486354

[26] Rouli L, MBengue M, Robert C, Ndiaye M, La Scola B, Raoult D. Genomic analysis of three African strains of *Bacillus anthracis* demonstrates that they are part of the clonal expansion of an exclusively pathogenic bacterium. New Microbe and New Infect. 2014;2:161–9. 10.1002/nmi2.62 PMC4265047 25566394

[27] Cohen-Gihon I, Israeli O, Beth-Din A, Levy H, Cohen O, Shafferman A, et al Whole-Genome Sequencing of the Nonproteolytic *Bacillus anthracis* V770-NP1-R Strain Reveals Multiple Mutations in Peptidase Loci. Genome Announc. 2014;2(1). Epub 2014/02/15. 10.1128/genomeA.00075-14 e00075-14 [pii]2/1/e00075-14 [pii]. 24526646 PMC3924378

[28] Birdsell DN, Antwerpen M, Keim P, Hanczaruk M, Foster JT, Sahl JW, et al Draft Genome Sequences of Two Bulgarian *Bacillus anthracis* Strains. Genome Announc. 2013;1(2):e0015213 Epub 2013/04/20. 10.1128/genomeA.00152-13 e00152-13 [pii]1/2/e00152-13 [pii]. 23599294 PMC3630405

[29] Khmaladze E, Birdsell DN, Naumann AA, Hochhalter CB, Seymour ML, Nottingham R, et al Phylogeography of *Bacillus anthracis* in the country of Georgia shows evidence of population structuring and is dissimilar to other regional genotypes. PLoS One. 2014;9(7):e102651 Epub 2014/07/23. 10.1371/journal.pone.0102651 PONE-D-13-55094 [pii]. 25047912 PMC4105404

[30] Blouin Y, Hauck Y, Soler C, Fabre M, Vong R, Dehan C, et al Significance of the identification in the Horn of Africa of an exceptionally deep branching *Mycobacterium tuberculosis* clade. PLoS One. 2012;7(12):e52841 Epub 2013/01/10. 10.1371/journal.pone.0052841 PONE-D-12-22563 [pii]. 23300794 PMC3531362

[31] Papazisi L, Rasko DA, Ratnayake S, Bock GR, Remortel BG, Appalla L, et al Investigating the genome diversity of *B*. *cereus* and evolutionary aspects of *B*. *anthracis* emergence. Genomics. 2011;98(1):26–39. Epub 2011/03/31. 10.1016/j.ygeno.2011.03.008 S0888-7543(11)00076-0 [pii]. 21447378 PMC3129444

[32] Price EP, Matthews MA, Beaudry JA, Allred JL, Schupp JM, Birdsell DN, et al Cost-effective interrogation of single nucleotide polymorphisms using the mismatch amplification mutation assay and capillary electrophoresis. Electrophoresis. 2010;31(23–24):3881–8. Epub 2010/11/11. 10.1002/elps.201000379 21064143 PMC3045815

[33] Fouet A, Smith KL, Keys C, Vaissaire J, Le Doujet C, Levy M, et al Diversity among French *Bacillus anthracis* isolates. J Clin Microbiol. 2002;40(12):4732–4. Epub 2002/11/28. 12454180 10.1128/JCM.40.12.4732-4734.2002 PMC154597

[34] Ohnishi N, Maruyama F, Ogawa H, Kachi H, Yamada S, Fujikura D, et al Genome Sequence of a *Bacillus anthracis* Outbreak Strain from Zambia, 2011. Genome Announc. 2014;2(2). Epub 2014/03/08. 10.1128/genomeA.00116-14 e00116-14 [pii]2/2/e00116-14 [pii]. 24604644 PMC3945500

[35] Hang'ombe MB, Mwansa JC, Muwowo S, Mulenga P, Kapina M, Musenga E, et al Human-animal anthrax outbreak in the Luangwa valley of Zambia in 2011. Trop Doct. 2012;42(3):136–9. Epub 2012/04/05. 10.1258/td.2012.110454 td.2012.110454 [pii]. . 22472314

[36] Lista F, Faggioni G, Valjevac S, Ciammaruconi A, Vaissaire J, le Doujet C, et al Genotyping of *Bacillus anthracis* strains based on automated capillary 25-loci Multiple Locus Variable-Number Tandem Repeats Analysis. BMC Microbiol. 2006;6(1):33 . 16600037 10.1186/1471-2180-6-33 PMC1479350

[37] Maho A, Rossano A, Hachler H, Holzer A, Schelling E, Zinsstag J, et al Antibiotic susceptibility and molecular diversity of *Bacillus anthracis* strains in Chad: detection of a new phylogenetic subgroup. J Clin Microbiol. 2006;44(9):3422–5. Epub 2006/09/07. 44/9/3422 [pii] 10.1128/JCM.01269-06 16954291 PMC1594716

[38] Jung KH, Kim SH, Kim SK, Cho SY, Chai JC, Lee YS, et al Genetic populations of *Bacillus anthracis* isolates from Korea. J Vet Sci. 2012;13(4):385–93. Epub 2012/12/29. 201212385 [pii]. 23271180 10.4142/jvs.2012.13.4.385 PMC3539124

[39] Quail MA, Smith M, Coupland P, Otto TD, Harris SR, Connor TR, et al A tale of three next generation sequencing platforms: comparison of Ion Torrent, Pacific Biosciences and Illumina MiSeq sequencers. BMC Genomics. 2012;13:341 Epub 2012/07/26. 10.1186/1471-2164-13-341 1471-2164-13-341 [pii]. 22827831 PMC3431227

[40] Calavas D, Sala C, Vaissaire J, Condé J, Thien-Aubert H, Hessemann M, et al Retour d'expérience sur un épisode de fièvre charbonneuse chez les bovins dans le Doubs au cours de l'été 2008. Bulletin épidémiologique. 2009;32:1–7.

[41] Morens DM. Characterizing a "new" disease: epizootic and epidemic anthrax, 1769–1780. Am J Public Health. 2003;93(6):886–93. Epub 2003/05/30. 12773345 10.2105/ajph.93.6.886 PMC1447860

[42] Morens DM. Epidemic anthrax in the eighteenth century, the Americas. Emerg Infect Dis. 2002;8(10):1160–2. Epub 2002/10/25. 10.3201/eid0810.020173 12396933 PMC2730311

