# Correction: Comparison of French and Worldwide *Bacillus anthracis* Strains Favors a Recent, Post-Columbian Origin of the Predominant North-American Clade

**DOI:** 10.1371/journal.pone.0180603

**Published:** 2017-06-27

**Authors:** Gilles Vergnaud, Guillaume Girault, Simon Thierry, Christine Pourcel, Nora Madani, Yann Blouin

The initially published S1 Table is missing some accession numbers necessary to retrieve raw sequence reads used to identity single nucleotide polymorphisms listed in S2 table. The revised version includes the full list of accession numbers together with links to facilitate the downloading of the associated data. More information for downloading from the European Nucleotide Archive (ENA) can be found at http://www.ebi.ac.uk/ena/.

CIP strains indexed 1 to 7 were initially obtained from the “Collection de Ressources Biologiques de l’Institut Pasteur” (CRBIP). We especially thank the CRBIP for the provision of these highly valuable historical strains.

## Supporting information

S1 Table List of strains used in this study. The geographic origin and year of isolation are indicated when known, together with the SNP lineage assignment (canSNP), accession numbers, sequencing level, and links to the corresponding data.
(XLSX)
